# Effect of individual preventive practices on COVID-19 infection: an analysis of big data collected at PCR testing centers in Hiroshima, Japan

**DOI:** 10.1186/s12889-025-21709-4

**Published:** 2025-02-19

**Authors:** Tatsuhiro Nagata, Odgerel Chimed-Ochir, Yui Yumiya, Junko Tanaka, Masao Kuwabara, Kanako Kitahara, Hiroki Ohge, Tatsuhiko Kubo

**Affiliations:** 1 Department of Public Health and Health Policy, Graduate School of Biomedical and Health Sciences, Hiroshima University, 1-2-3 Kasumi, Minami-ku, Hiroshima, 734-8553 Japan; 2 Medical Policy Office, Hiroshima University, 1-2-3 Kasumi, Minami-ku, Hiroshima, 734-8553 Japan; 3 Hiroshima Prefectural Center for Disease Control and Prevention, 10-52, Motomachi, Naka-ku, Hiroshima-shi, Hiroshima-ken, Hiroshima, 730-8511 Japan; 4 Hiroshima Prefectural Government Health and Welfare Affairs Bureau, 10-52, Motomachi, Naka-ku, Hiroshima-shi, Hiroshima-ken, Hiroshima, 730-8511 Japan; 5 Department of Infectious Diseases, Hiroshima University Hospital, 1-2-3 Kasumi, Minami-ku, Hiroshima, 734-0037 Japan

**Keywords:** COVID-19, Public health, Behavioral risk factor surveillance system, Public health surveillance, Prevention and control, NPIs, J-SPEED, Japan

## Abstract

**Background:**

By May 7, 2023, COVID-19 had significantly impacted Japan, with 33,728,909 infections and 74,663 deaths reported. Hiroshima Prefecture alone recorded 816,354 cases and 1,373 deaths. The World Health Organization emphasized the importance of non-pharmaceutical interventions (NPIs) for preventing infectious disease transmission. Individual NPIs, such as hand hygiene, mask wearing, and avoiding crowded places, comprise simple everyday measures that individuals can personally undertake to protect themselves and others from contracting and transmitting respiratory infections. Japan’s Cabinet Secretariat also recommended these measures. Previous studies investigated the effectiveness of NPIs but often used relatively short data-collection periods. Starting in May 2020, Hiroshima Prefecture adopted a unique COVID-19 public health surveillance policy that used standardized data-collection forms. The present analysis examines the association between individual NPIs and COVID-19 infections.

**Methods:**

Data were collected at 14 PCR centers from April 1, 2021, to August 3, 2022 in Hiroshima Prefecture. Participants filled out the J-SPEED-style COVID-19 form, which included items on demographic information, job type, symptoms, and NPIs. The data were analyzed for demographic information, NPI compliance rates, infection rates in relation to NPI adoption, and adjusted risk ratios, which were obtained using a multivariate log-binomial regression model.

**Results:**

A total of 1,125,188 tested cases from 4th to 7th waves were analyzed. Among the study population, the infection rate increased through the various waves, with the highest rate (8.3%) seen in the 7th wave. Adults aged 40–49 were most commonly tested, while those aged 60–69 had the lowest infection rates. Wearing masks/washing hands was the most commonly followed NPI. Compliance with NPIs decreased through the waves. Individuals adhering to NPIs had lower infection rates. The number of preventive measures adopted was correlated with a reduced infection risk.

**Conclusion:**

This analysis provides evidence to guide COVID-19 prevention policies. Simultaneous adherence to multiple NPIs proved more effective in preventing COVID-19. Despite changes in viral strains and the number of infected cases, hand washing/mask wearing, refraining from travel, and refraining from dining out significantly associated with a reduction of COVID-19 infection. Our findings are likely to be applicable in future infectious disease outbreaks.

## Background

Between the onset of COVID-19 in 2019 and May 7, 2023, the disease impacted a significant number of individuals, with 33,527,813 infections and 74,654 deaths recorded in Japan. In Hiroshima Prefecture, the toll stood at 816,354 confirmed cases and 1,373 reported deaths [1].

The World Health Organization (WHO) has published guidelines outlining the implementation of individual and social non-pharmaceutical interventions (NPIs) as a crucial strategy for preventing and controlling the transmission of infectious diseases, including but not limited to COVID-19 [2]. NPIs encompass a range of actions that individuals and communities can adopt to effectively curtail the spread of contagious diseases, such as pandemic flu or COVID-19. These interventions are typically categorized into two main types: individual NPIs and social NPIs [2]. Individual NPIs primarily comprise simple, everyday measures that individuals can personally undertake to protect themselves and others from contracting and transmitting respiratory infections [3]. These measures often include practices such as proper hand hygiene, mask wearing, maintaining physical distance, and avoiding crowded places. The WHO urged all Member States to encourage their populations to embrace these preventive measures as a collective responsibility [4].

Japan’s Cabinet Secretariat also introduced comprehensive guidelines highlighting essential infection control measures, including a range of individual NPIs. These guidelines stressed the importance of measures such as mask wearing, regular room ventilation, and frequent hand washing as a part of collective effort to combat the spread of the virus [5].

Previous studies investigated the effectiveness of individual NPIs across various countries [6–8] using diverse methodologies, including questionnaire surveys [7, 8] and simulation modeling approaches [8, 9]. However, the prior questionnaire surveys involved relatively short data-collection periods.

To our knowledge, no prior study has sought to comprehensively collect and analyze epidemiological data spanning beyond a year. Furthermore, no study has sought to thoroughly compare multiple infection waves to comprehensively assess the effectiveness of preventive measures. Here, we aimed to bridge this gap by examining an extended timeframe and holistically assessing prevention effectiveness across various infection waves.

Beginning in May 2020, Hiroshima Prefecture in Japan implemented a distinctive COVID-19 public health surveillance policy through which standardized data collection forms known as the Japan Surveillance in Post Extreme Emergencies and Disasters (J-SPEED) were used to gather information from various sources, including public health centers, recuperation hotels, online treatment centers, oxygen centers, PCR testing centers, and hospitals. Here, we investigated the association between prevention behaviors and COVID-19 infection using data collected at PCR testing centers from 1 April 2021 to 3 August 2022.

## Materials and methods

### Data collection

In Hiroshima Prefecture, Japan, 14 PCR centers were established to provide free testing access for people living in and traveling to Hiroshima Prefecture. Upon visiting a center, each individual was asked to complete a pre-administered questionnaire known as the J-SPEED -style COVID-19 form. The original J-SPEED form was initially developed to be used during natural disasters. Based on lessons learned from the Great East Japan Earthquake in 2011, the form was designed to simplify and standardize health data and enable its nearly real-time collection. An adapted form was used for COVID-19 public health surveillance in Hiroshima Prefecture [10]. 

The PCR center version of the J-SPEED-style COVID-19 form (hereinafter called J-SPEED COVID-19) underwent multiple revisions; it originally had 40 items, but the two versions used for this analysis included 40 and 46 items. The items included information on demographics, job types, symptoms, existing diseases, vaccination status, COVID-19 preventive measures and PCR test results.

Data collection at PCR centers started on 1 April 2021 and ended on 7 May 2023. However, we used data from the 4th through 7th waves of COVID-19 infection, spanning 1 April 2021 to 3 August 2022. The data were cleaned, and out of 1,127,526 cases that received testing at a PCR center, a total of 1,125,188 cases were analyzed. The study period was divided into four waves, as follows: 4th wave (2021/4/1–2021/6/30), 5th wave (2021/7/1–2021/11/30), 6th wave (2021/12/1–2022/6/30), and 7th wave (2022/7/1–2022/8/3), as recommended by Hiroshima Prefecture [11].

In the PCR center version of the J-SPEED-style COVID-19 form, four main preventive measures were included: wearing masks and washing hands, refraining from dining outside, refraining from traveling, and telework. These questions were designed to determine whether participants generally engaged in specific NPIs, such as mask-wearing and handwashing, without delving into the frequency or specific contexts of these behaviors. The first three measures —mask-wearing, handwashing, and refraining from dining and traveling— are typically voluntary and directly controlled by individuals. In contrast, telework is often determined by employment conditions and the feasibility within specific occupational contexts. This distinction means that telework does not align seamlessly with the individual-focused preventive measures. Consequently, we excluded teleworking from our analysis to maintain a clear focus on behaviors that are directly within individuals’ control. If the PCR test result was positive, the subject was deemed “infected,” otherwise they were deemed “non-infected”.

### Data analysis

First, we examined the demographic information of the study participants. Second, for each of the four waves, we computed the compliance rates for individual preventive measures and assessed their trends, utilizing the Cochrane Armitage test. Third, we calculated the infection rates in relation to the adoption of preventive measures. Lastly, the adjusted risk ratios were calculated to examine the risk discrepancy between individuals who adhered to the studied preventive measures and those who did not. Given that our outcome variable was binary (infected or non-infected), we employed a multivariate log-binomial regression model [12, 13]. This model was adjusted for age, sex, other preventive measures, and exposure to close contacts within a 14-day period. Vaccine data became available from December 2021; therefore, vaccination status was adjusted for the model only in waves 6 and 7. The Proc GenMod procedure of the SAS software program was used to run log-binomial regression [14]. The results of the multivariate log-binomial regression model were visually displayed in a forest plot.

EZR (ver.1.55) [15] and SAS Version 9.4 (SAS Institute, Inc., Cary, NC, USA) were used for data analysis. Approval for ethical review was obtained from Hiroshima University (approval number: E-2508).

## Results

Table 1 shows the general characteristics of the study participants and their infection rates from the 4th to 7th waves of COVID-19 in Hiroshima prefecture. Throughout the study period, 1,125,188 people received testing at a PCR center; of them, 3.3.% were infected with COVID-19. The infection rate increased through the waves, with the lowest infection rate seen in the 4th wave (0.5%) and the highest infection rate seen in 7th wave (8.3%). In terms of sex and pregnancy status, pregnant women had a highest infection rate (4.3%). Children aged 0 to 9 years had the highest overall infection rate (6.5%), whereas those aged 60–69 years had the lowest overall infection rate (2.1%). The same age-related tendencies in infection rate were seen for the 6th and 7th waves, whereas during the 4th and 5th waves those aged 20 to 29 years had higher infection rates.

**Table 1:** Demographic characteristics and COVID-19 infection rate among study participants

	Entire period			4th Wave			5th Wave			6th Wave			7th Wave		
	N	N of infection cases	Infection rate	N	N of infection cases	Infection rate	N	N of infection cases	Infection rate	N	N of infection cases	Infection rate	N	N of infection cases	Infection rate
Covering period	2021/4/1 - 2022/8/3			2021/4/1 - 2021/6/30			2021/7/1 - 2021/11/30			2021/12/1 - 2022/6/30			2022/7/1 - 2022/8/3		
Total	1E+06	37,414	3.3%	264,320	1229	0.5%	237,087	2039	0.9%	521,145	25,612	4.9%	102,636	8534	8.3%
Sex															
Male	594,700	20,476	3.4%	144,499	690	0.5%	127,300	1331	1.0%	270,503	13,810	5.1%	52,398	4645	
Female Non-pregnant	519,999	16,575	3.2%	116,866	525	0.4%	107,234	690	0.6%	246,470	11,558	4.7%	49,429	3802	8.9%
Female Pregnant	8198	352	4.3%	1644	8	0.5%	1628	13	0.8%	4117	244	5.9%	809	87	7.7%
Age															10.8%
0-9	51,569	3333	6.5%	4138	12	0.3%	7749	66	0.9%	33,111	2525	7.6%	6571	730	
10-19	109,816	4834	4.4%	16,156	95	0.6%	25,691	199	0.8%	57,054	3567	6.3%	10,915	973	
20-29	184,736	7018	3.8%	42,083	421	1.0%	45,281	650	1.4%	82,265	4690	5.7%	15,107	1257	11.1%
30-39	169,044	6780	4.0%	38,733	207	0.5%	35,705	363	1.0%	79,088	4637	5.9%	15,518	1573	8.9%
40-49	214,557	6661	3.1%	52,977	195	0.4%	44,973	352	0.8%	97,102	4516	4.7%	19,505	1598	8.3%
50-59	193,191	4322	2.2%	50,797	124	0.2%	41,799	247	0.6%	83,798	2809	3.4%	16,797	1142	10.1%
60-69	125,450	2619	2.1%	35,892	105	0.3%	24,435	107	0.4%	54,147	1639	3.0%	10,976	768	8.2%
over70	76,570	1845	2.4%	23,339	70	0.3%	11,434	55	0.5%	34,555	1227	3.6%	7242	493	6.8%

Table 2 depicts the proportion of study participants using the various preventive measures across the four distinct waves, along with the corresponding trends. Wearing masks/washing hands were the most widely followed preventive measure throughout the four waves. Over the course of these waves, the proportion of individuals who reported wearing masks and hand washing declined. Similarly, there was a decline in the proportion of people refraining from travel and dining out during these waves. The percentage of individuals who reported simultaneously adhering to three preventive measures, including masks/hand washing, refraining from traveling, and refraining from dining out, decreased from 52.0% during the 4th wave to 41.1% during the 7th wave.

**Table 2:** Proportion of study participants using the various preventive measures

	4th Wave			5th Wave			6th Wave			7th Wave			Trend analysis	
	N of people tested	N of people taken prevention	% of people taken prevention	N of people tested	N of people taken prevention	% of people taken prevention	N of people tested	N of people taken prevention	% of people taken prevention	N of people tested	N of people taken prevention	% of people taken prevention	Slope	P for trend
**Types of prevention**														
Refraining from travel	264,320	177,777	67.3%	237,087	147,405	62.2%	521,145	329,976	63.3%	102,636	59,037	57.5%	-0.023	*P*<0.001
Refraining from eating out of home		179,719	68.0%		155,257	65.5%		323,411	62.1%		53,907	52.5%	-0.041	*P*<0.001
Wearing face mask and Proper hand washing		243,287	92.0%		213,993	90.3%		474,935	91.1%		92,783	90.4%	-0.004	*P*<0.001
**Number of prevention**														
None	264,320	6796	2.6%	237,087	9330	3.9%	521,145	17,860	3.4%	102,636	3845	3.7%	0.003	*P*<0.001
One		51,584	19.5%		54,364	22.9%		137,147	26.3%		34,052	33.2%	0.040	*P*<0.001
Two		68,621	26.0%		57,888	24.4%		107,239	20.6%		22,542	22.0%	-0.021	*P*<0.001
Three		137,319	52.0%		115,505	48.7%		258,899	49.7%		42,197	41.1%	-0.021	*P*<0.001

Table 3 presents the infection rates in relation to preventive measures. Individuals who consistently adhered to wearing masks/washing hands, refraining from travel, and refrained from eating out exhibited lower infection rates than those who didn’t follow these measures. Infection rates showed a steady decline in correlation with the number of adopted preventive measures across all four waves.

**Table 3:** COVID-19 infection rate in relation to prevention measures

	4th Wave				5th Wave				6th Wave				7th Wave			
	*N* of people taken prevention	Infection rate	*N* of people not taken prevention	Infection rate	*N* of people taken prevention	Infection rate	*N* of people not taken prevention	Infection rate	*N* of people taken prevention	Infection rate	*N* of people not taken prevention	Infection rate	*N* of people taken prevention	Infection rate	*N* of people not taken prevention	Infection rate
**Types of prevention**																
Refraining from travel	177,777	0.42%	86,543	0.55%	147,405	0.79%	89,682	0.98%	329,976	4.65%	191,169	5.37%	59,037	7.83%	43,599	8.97%
Refraining from eating out of home	179,719	0.35%	84,601	0.71%	155,257	0.70%	81,830	1.17%	323,411	4.40%	197,734	5.75%	53,907	7.70%	48,729	8.99%
Wearing face mask and Proper hand washing	243,287	0.44%	21,033	0.76%	213,993	0.81%	23,094	1.36%	474,935	4.81%	46,210	6.02%	92,783	8.10%	9853	10.31%
**Number of prevention**																
None	6796	0.78%	NA		9330	1.76%	NA		17,860	7.95%	NA		3845	11.91%	NA	
One	51,584	0.69%			54,364	1.13%			137,147	5.61%			34,052	9.11%		
Two	68,621	0.53%			57,888	0.74%			107,239	4.45%			22,542	7.67%		
Three	137,319	0.33%			115,505	0.72%			258,899	4.53%			42,197	7.69%		

**Fig. 1:**
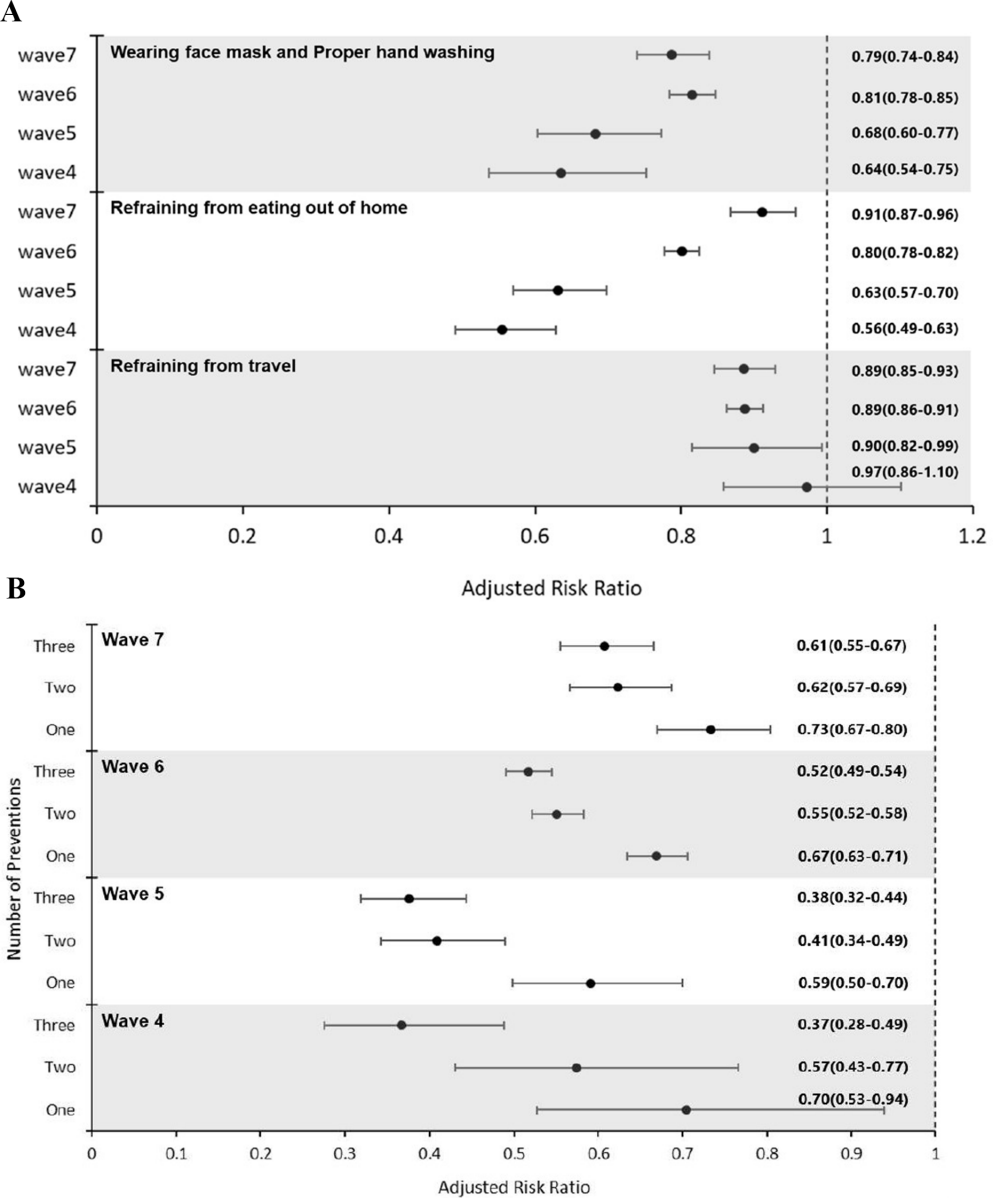
(**A**). Association between adherence to preventive measures and COVID-19 infection rates. (**B**). Association between number of adhered-to preventive measures and COVID-19 infection rates

Figure 1 depicts the relationship between infection rates and the adherence to preventive measures (Fig. 1A) and number of preventive measures taken simultaneously (Fig. 1B), expressed as risk ratios. The figure conveys three key points: (i) Across all waves, individuals who adhered to wearing masks/practicing hand washing and those who refrained from eating out consistently exhibited a significantly lower risk of contracting COVID-19 compared to those who did not adopt these measures. Additionally, during the 5th and 7th waves, individuals who refrained from travel had a significantly reduced risk of infection compared to those who didn’t follow this measure. (ii) Observation of changes in risk ratios across the four waves revealed that the risk ratios increased with the wave number for individuals practicing mask wearing/hand washing, as well as those refraining from eating outside. (iii) During all four waves, the risk of infection showed a consistent decrease in parallel with the increase in the number of preventive measures adopted.

## Discussion

This research aimed to investigate the impact of three basic individual non-pharmaceutical preventive measures on COVID-19 infection, namely wearing masks/washing hands, refraining from travel, and refraining from eating outside. Throughout the four studied waves of infection in Hiroshima prefecture, Japan, the average rate of mask wearing/hand washing exceeded 90%.

Japan stands out as having maintained high mask usage rates compared to those of other countries [16]. Several factors contribute to this widespread adoption of masks in Japan. Even long before the COVID-19 pandemic, wearing masks had been ingrained in Japanese culture as a means to protect against colds and hay fever [16]. As a result, people were more inclined to persist in using masks throughout the pandemic. Furthermore, a strong sense of social responsibility prevails in Japan and wearing masks is viewed as a way to safeguard others from falling sick [16]. Although the Japanese government withdrew its official recommendation to wear masks on March 13, 2023 [17], allowing individuals to make their own decisions in this regard, many people continue to wear masks voluntarily in Japan [18, 19].

Our study showed that as the wave number increased there was a small but statistically significant decrease in compliance with mask usage/hand washing, refraining from dining outside, and refraining from travel. This time-dependent decline in adherence to protective measures is commonly referred to as “COVID-19 fatigue” [20–22] and is believed to reflect the weariness that individuals experienced concerning ongoing precautionary measures and the persisting threat of COVID-19 [23]. The consequences of pandemic fatigue can manifest in various ways, with individuals feeling burnt out, fatigued, and demotivated to continue engaging in protective behaviors [21]. Previous studies found gradual reductions in adherence to protective behaviors against COVID-19 from March through December 2020, aligning with the expectation that fatigue sets in over time [22].

The main finding of this study was that individuals who adhered to the studied preventive measures had a lower risk of being infected with COVID-19 compared to those who did not follow these measures.

The correlation between the implementation of wearing masks/practicing good hand washing and a reduced infection rate of COVID-19 is well-supported by multiple studies [16, 23–25]. These simple yet crucial preventive actions have been widely recommended by health authorities to curb the spread of infections, including COVID-19 [26].

Additionally, restrictions on risky behaviors, such as eating out at restaurants and traveling between prefectures, have been identified as potentially effective strategies in controlling the spread of infectious diseases in other studies [16, 23, 24, 27–30]. Limiting non-essential activities and mobility can help reduce opportunities for the virus to spread and minimize contact among individuals. In line with these findings, infectious disease experts have stated that dining at restaurants, both indoors and outdoors, can pose a moderate-to-high risk of COVID-19 transmission [31].

Given the consensus across multiple studies and expert opinions, it is evident that preventive measures like wearing masks, maintaining proper hand hygiene, and implementing restrictions on certain activities can play crucial roles in controlling the spread of infectious diseases, particularly during pandemics like COVID-19. Therefore, it is essential to continue promoting these practices to safeguard public health and prevent further outbreaks.

Our finding also revealed that the preventive power of practices such as mask wearing/hand washing and refraining from eating outside of the home appeared to decrease as each new wave appeared. There could be several potential explanations for this phenomenon. First, the new viral variants involved in successive waves were more transmissible than previous variants and could have evaded some level of protection conferred by compliance to preventive measures. This aligns with the observation that our study’s timeframe mirrors the emergence and spread of the Omicron variant, which was identified as the most transmissible among the variants, followed by the Delta, Alpha, Gamma, and Beta variants [32]. Additional explanations could involve inadequate compliance with preventive measures and/or misreporting. As mentioned above, COVID-19 fatigue could mean that individuals may have failed to wear masks consistently or correctly yet reported full compliance with preventive measures. Third, even individuals who diligently followed preventive measures could have become infected due to extended close contact with infected people.

Our findings indicate that implementing multiple preventive measures simultaneously is associated with a greater reduction in COVID-19 cases compared to not implementing any measures. Therefore, by combining different measures, policymakers can create a multi-layered defense against a virus to increase the likelihood of successfully controlling its spread. However, it would be essential for policymakers to continuously monitor the situation, evaluate the effectiveness of the utilized measures, and make data-driven adjustments as needed. In addition, collaboration between policymakers, health authorities, and the public is crucial to ensure the effective implementation of these strategies and protect communities from the ongoing impact of the pandemic. In the current study, the implementation of a simple data collection form known as J-SPEED proved to be highly advantageous, as it facilitated effective and timely data reporting and feedback to local authorities. This successful experience suggests that J-SPEED could be a valuable tool for enhancing infectious disease control measures in the future.

The current study has strengths and weaknesses. This study’s advantages lie in its utilization of large-scale and long-term data collection, which allowed for comparisons among four waves of infection that occurred in Hiroshima Prefecture. Moreover, the prefecture-level management of PCR testing ensured that the collected data was highly accurate. The study’s focus on both positive cases and negative individuals provides a more comprehensive understanding of preventive actions. Despite its strengths, however, this study also has several limitations that warrant consideration. One significant limitation pertains to the use of a self-administered questionnaire that lacked any means of externally validating the provided answers. Thus, it was challenging to ascertain whether participants had been consistently (versus sporadically) following the preventive measures to which they reported adhering. Consequently, our present results may underestimate the true impact of preventive measures on COVID-19 transmission. Another potential limitation is that, due to the constraints of the emergency setting and the need for rapid data collection, our survey focused on whether participants generally engaged in specific individual interventions, such as mask-wearing and handwashing, without assessing the frequency or specific contexts of these behaviors. This approach may have introduced a certain level of inaccuracy into our analysis. Regarding mask-wearing and handwashing, it is true that these two NPIs serve different primary purposes—mask-wearing primarily mitigates respiratory droplet transmission, while handwashing targets fomite transmission and general hygiene. However, these interventions were combined in the questionnaire to align with the guidelines provided by the Ministry of Health and Welfare of Japan [33], which promoted mask-wearing and handwashing as integrated preventive practices during the COVID-19 pandemic. Consequently, the effects of each intervention could not be analyzed separately. Given that these interventions have distinct purposes and implications, future studies should consider analyzing them individually to provide more nuanced insights.

Furthermore, certain important factors were not accounted for in the analysis, such as whether participants had family members living with them. These factors could significantly influence the risk of infection. To provide a more comprehensive understanding of the data and a more nuanced analysis, future studies must incorporate and consider these factors when examining the relationship between preventive measures and COVID-19 transmission.

Generalizing the results to other regions or countries should be done with caution, as cultural variations may significantly impact the effectiveness of certain preventive measures. Therefore, further research using data from overseas is needed to enhance the applicability and generalizability of the present findings.

## Conclusions

Our analysis supports evidence that could be used in drafting policies on COVID-19 infection prevention. Specifically, three preventive measures of hand washing/mask wearing, refraining from travel, and refraining from dining out were significantly associated with a reduction in COVID-19 infection rates, even though their compliance decreased over time. The epidemiological findings of this study suggest that a certain level of these prevention actions is associated with mitigating COVID-19 transmission, despite changes in coronavirus strains and the number of infected persons. Furthermore, these measures are likely to remain supportive in controlling future outbreaks of other respiratory infectious diseases.

## Data Availability

In accordance with the Ethical Committee protocol, the data must be kept at the Department of Public Health and Health Policy, Hiroshima University, Japan and are not to be shared without permission of Hiroshima Prefecture, Japan. Therefore, the data shall only be made available with permission of Hiroshima Prefecture for reasonable purposes. Please initiate contact through tkubo@hiroshima-u.ac.jp to request the permission.
